# Semi-supervised exercise training program more effective for individuals with postural orthostatic tachycardia syndrome in randomized controlled trial

**DOI:** 10.1007/s10286-023-00970-w

**Published:** 2023-08-20

**Authors:** Courtney M. Wheatley-Guy, Meredith G. Shea, Jordan K. Parks, Robert Scales, Brent P. Goodman, Richard J. Butterfield, Bruce D. Johnson

**Affiliations:** 1 Division of Cardiovascular Diseases, Mayo Clinic, 13400 East Shea Blvd, Scottsdale, AZ 85259 USA; 2 Department of Neurology, Mayo Clinic, Scottsdale, AZ USA; 3 Department of Quantitative Health Sciences, Mayo Clinic, Scottsdale, AZ USA

**Keywords:** Cardiopulmonary exercise testing (VO_2_), Personalized exercise program, Exercise tolerance, COMPASS 31

## Abstract

**Purpose:**

Exercise like any medication requires the correct dose; to be effective the appropriate frequency, duration, and intensity are necessary. This study aimed to assess if a semi-supervised exercise training (ET) program would be more effective at improving aerobic fitness (VO_2PEAK_), exercise tolerance, and symptoms in individuals with postural orthostatic tachycardia syndrome (POTS) compared to the standard of care (SOC).

**Methods:**

Subjects were randomized to either the ET or SOC groups (*n* 26 vs. 23; age 33 ± 11 vs. 37 ± 10 years; VO_2PEAK_ 66 ± 15 vs. 62 ± 15% predicted, ET vs. SOC respectively, *p* > 0.05). Composite Autonomic Symptom Score (COMPASS 31), 10 min stand test, and cardiopulmonary exercise test were performed at baseline and following 12 weeks. The ET group received an exercise consultation and eight semi-supervised in-person or virtual exercise sessions.

**Results:**

The ET group demonstrated a greater improvement in VO_2PEAK_, higher or longer tolerance for baseline peak workload, and more often had a delayed symptom onset with exercise than the SOC group (ΔVO_2PEAK_ 3.4 vs. − 0.2 mL/min/kg, *p* < 0.0001, ΔWorkload 19 ± 17 vs. 0 ± 10 W; Workload time 63 ± 29 vs. 22 ± 30 s; onset-delay 80% vs. 30%, *p* < 0.05). Individuals in the ET group reported a significant improvement in orthostatic intolerance domain score (*p* = 0.02), but there was not a significant difference in the improvement in total COMPASS score (− 11.38 vs. − 6.49, *p* = 0.09).

**Conclusion:**

Exercise training was more effective with greater improvements in aerobic fitness, orthostatic symptoms, and exercise tolerance for individuals with POTS when intensity and progression were personalized and delivered with minimal supervision compared to the SOC.

**Supplementary Information:**

The online version contains supplementary material available at 10.1007/s10286-023-00970-w.

## Introduction

Previous research has shown that patients with postural orthostatic tachycardia syndrome (POTS) experience exercise intolerance and are more deconditioned than their sedentary, but otherwise healthy counterparts [1–5]. Additionally, it is clear that the act of limiting physical activity to avoid symptoms can actually aggravate the condition by reducing myocardial work and causing consequential ventricular remodeling, which has been seen with prolonged bed rest [6, 7]. Further, there is an inverse relationship between heart rate and stroke volume highlighting that although cardiac output can be maintained, it results in elevated heart rates as a compensation for reductions in stroke volume [7, 8]. Work by Masuki et al. compared cardiac response to supine and upright exercise between POTS and healthy sedentary controls (removing deconditioning component) and found that individuals with POTS had a greater elevation in heart rate during matched workloads when in both the upright and supine positions compared to sedentary controls. This was secondary to a reduced stroke volume which was significantly more pronounced during upright exercise in individuals with POTS [8]. Although not the cause, the majority of patients with POTS are at least mildly deconditioned which can influence the severity of symptoms and is something that is treatable. Previous research has demonstrated that a 3-month exercise training program can help treat the cardiac origins of the condition by increasing blood volume, left ventricular mass, and consequently stroke volume to improve aerobic fitness [9]. Most importantly starting and maintaining an active lifestyle can be the key to reducing or eliminating symptoms [10]. 

“Exercise is medicine” for almost all conditions including POTS [11], but what is currently lacking is an effective way to deliver an exercise prescription and assist patients with its implementation. The current standard of care for individuals with POTS targets multiple aspects of the pathology and includes recommendations for physical therapy and/or aerobic exercise. The recommendation is a graded exercise regimen building to a target of 30 min, 5 days a week starting with horizontal or recumbent exercise modalities, but no personalized and progressive exercise training program or supervised instruction is provided. Even for healthy individuals there can be hesitation, anxiety, and unfamiliarity when starting an exercise program. This is amplified for those with POTS or other conditions who worry how to exercise safely given risk of post-exertional malaise or symptom exacerbation. Although for some this recommendation will be sufficient, the majority will not be successful in implementing and achieving 150 min of activity/week for multiple reasons. In the study exercise training was delivered in a semi-supervised manner either in-person or virtually.

The goal of this study was to determine if a semi-supervised and personalized exercise training program could provide a more effective way to achieve improvements in aerobic fitness, functional ability, and reduce disease severity or symptoms for individuals with POTS than the current standard of care. The secondary aim was to assess the efficacy of a semi-supervised exercise training program on adherence, achieving the target intensity, and improving exercise tolerance in individuals with POTS.

## Methods

### Study design

Subjects completed a study visit at baseline and after the 3-month intervention period. During each study visit subjects completed a 10-min stand test and questionnaires to assess orthostatic tolerance and symptom severity, 24 h urine sodium, bioimpedance assessment of fluid, and a maximal exercise test. Following the baseline visit, subjects were randomized to either the exercise training (ET) or standard of care (SOC) groups using a blocked randomization scheme stratified on the basis of hypermobile Ehlers–Danlos syndrome (hEDS) diagnosis and equal randomization between groups every four subjects. Participants returned for a 12-week follow-up visit where all testing done on the baseline visit was repeated. The Mayo Clinic Institutional Review Board reviewed and approved the protocol. All participants provided written informed consent prior to study and all aspects of the study were performed in accordance with the declaration of Helsinki.

### Subjects

Sixty individuals diagnosed with POTS by positive tilt table or autonomic reflex screen where they had met diagnostic criteria for POTS with heart rate increase of 30 beats per minute (bpm) or more, or over 120 bpm, within the first 10 min of standing or assuming an upright posture, in the absence of orthostatic hypotension and had been experiencing chronic symptoms of orthostatic intolerance for at least 6 months agreed to participate in this study. Exclusion criteria included (1) pregnancy or (2) regularly active (more than 30 min/week of structured moderate intensity aerobic exercise). Subjects were randomized to either the ET or SOC groups. Patients with hypermobile hEDS were eligible to participate, but because these patients have their own unique challenges regarding exercising, randomization was also stratified on the basis of whether the individual had hEDS or not to ensure equal distribution of those who had both POTS and hEDS between the ET and SOC groups. Classification of hEDS was confirmed by a documented clinical diagnosis; if there was no clinical diagnosis, participants who reported hypermobility were classified as having hypermobility spectrum disorder. All participants were asked to not take any medication that can affect their autonomic system for more than 24 h if feasible, if not they were evaluated under identical conditions (*n* = 5, took same medications prior to both visits).

### Evaluation of symptom improvement

Tolerance of positional changes was evaluated by having participants stand for 10 min, or as long as tolerated, following a 30-min supine period (10 min stand test) while heart rate was continuously monitored and blood pressure assessed at 1, 3, 5, and 10 min of standing. Subjects completed the Composite Autonomic Symptoms Questionnaire (COMPASS 31) used to assess the presence and severity of symptoms. The Short Form 36 Health Questionnaire (SF-36) was completed to assess quality of life and functional health/well-being reviewing physical component and mental component summary scores. Subjects rated their functional ability using a 0–100% scale categorized into 10% increments with 0% describing being completely bed ridden and 100% being able to work full-time with no modifications. This scale can be reviewed in the supplemental material and was used previously by Schofield and Chemali [12].

### Evaluation of aerobic fitness, exercise tolerance, and symptom onset/severity

Subjects arrived in a 2-h fasted state. Following the evaluation of symptoms, subjects were then outfitted with a 12-lead electrocardiogram (ECG) (GE Healthcare, Chicago, IL) and forehead pulse oximeter (Masimo, Irvine, CA) to allow for continuous monitoring of heart rhythms and heart rate and peripheral oxygen saturation. The symptom-limited maximal exercise test was performed on a cycle ergometer (Corvial Lode B.V., the Netherlands). Exercise protocol was a 2-min warm up at 12 W, 3 min at the matched workload of 25 W, and then wattage increased every 2 min by 25 W. The test was continued until the subject’s symptoms worsened forcing them to terminate exercise or exhaustion was reached. Cuff auscultation midway through each exercise stage by the same technician assessed blood pressure. Respiratory gas exchange including oxygen uptake (VO_2_) was monitored continuously (MGC Diagnostics, St. Paul, MN) and averaged over the last 30 s of the stage for reported values. Peak oxygen consumption (VO_2PEAK_) is the maximal rate of oxygen consumption measured during exercise and provides an assessment of aerobic fitness. The highest 10-s average oxygen consumption achieved during the final stage of exercise was recorded as VO_2PEAK_ for each subject. Predicted VO_2PEAK_ was calculated according to the Mayo Clinic Cardiovascular Health Clinic equation: Male = (58.5 − age × 0.44) × 0.9 and Female = (45.3 − age × 0.37) × 0.9*.* Peak VO_2_ was then used to identify baseline aerobic fitness or deconditioning.

In addition to rating perceived exertion (RPE) using the Borg Perceived Exertion Scale and dyspnea using the Modified Borg Dyspnea Scale [13], subjects rated their symptom severity at baseline and each stage of exercise using the Buffalo Concussion Treadmill Test (BCTT) symptom severity 0–10 scale [14]. A copy of the BCTT symptom scale is provided in the supplemental material.

### Assessment of cardiac output and oxygen pulse

Measurement of cardiac output was performed in triplicate at rest and duplicate during exercise at matched 25 W and anaerobic threshold (AT) with the subjects in an upright seated position on the cycle ergometer using a rebreathing technique described previously [15–18]. Briefly, using a 5-L anesthesia bag containing 0.7% acetylene, 9% helium, and 35% O_2_ at a respiratory rate of 32 breaths/minute at the end of a normal expiration, the subjects were switched into the rebreathe bag and completed 6–10 consecutive breaths out of the bag. Cardiac output was not measured at peak as the rebreathe maneuver can be uncomfortable, and we did not want to increased dyspnea during the maneuver and cause early exercise termination. The rate of disappearance of acetylene from the exhaled gas mixture during rebreathing provides an assessment of pulmonary blood flow. Since acetylene does not bind to hemoglobin, the rate of disappearance of acetylene provides a reliable measure of cardiac output and has previously been validated in our laboratory using direct Fick during exercise [15]. Oxygen pulse is the ratio of oxygen consumption over heart rate and based on the Fick equation reflects the amount of oxygen consumed per heartbeat (i.e., stroke volume multiplied by arteriovenous oxygen difference [C(a–v) O2)].

#### Intervention

The SOC group followed the recommendations of their primary neurologist or cardiologist for managing the treatment of their symptoms which could include recommendations to increase salt and water intake, physical therapy, medications, and aerobic exercise. One participant in the SOC group had a medication change during the intervention period. Two in this group received an exercise consultation and the same exercise program, but no supervised exercise sessions. One completed physical therapy.

The ET group received an in-person exercise consultation and eight supervised in-person or virtual exercise sessions (weekly for the first month and biweekly for the remaining 2 months). Participants completed three aerobic sessions/week, targeting 20–30 min of interval training which started with short durations (3 min) and progressed by increasing working interval and shortening recovery intervals eventually progressing to 30 min of continuous exercise at a moderate intensity. Modality of exercise started on semi-recumbent modalities for most and progressed to upright modalities (upright bike or treadmill) as tolerated. Target heart rate training zones were provided on the basis of the peak heart rate and symptom onset during the maximal exercise test. The general recommendation was to perform the working interval at 75–85% of the individual’s peak heart rate, but monitoring breathing and symptoms were to be the primary criteria used in selecting desired intensity. Participants in the ET group were asked to not make any modifications to their POTS-related medications during the study period, and all complied.

Subjects in both groups were asked to track all structured exercise using a chest strap heart rate monitor (Polar, Bethpage, NY) and mobile app (24aLife, Rochester, MN). The research team reviewed each participant’s tracked sessions. An activity load for each session was calculated using the session total duration in minutes and average heart rate for each session as a percentage of the participant’s peak heart rate from the maximal exercise test. The activity load equaled session duration × (average HR/peak heart rate from baseline exercise test). A total activity load for all sessions over 12 weeks and average weekly activity load over the 3-month period were then calculated. This allowed for each session to be assessed on the basis of all factors of activity load (duration, intensity, and frequency) and to make it personalized on the basis of each individual’s measured maximum heart rate. For example, if an individual exercised for 60 min at 50% of their maximum heart rate the activity load would be 30 min, compared to an individual who exercised for only 45 min at 80% of their maximum heart rate where the activity load would be 36 min.

### Statistical analysis

All baseline characteristics including age, sex, and POTS status (severity and length of diagnoses) were compared for the SC and ET arms using Kruskal–Wallis test for continuous variables and Fisher’s exact test for categorical variables. Significant differences between ET and SOC groups were not observed; therefore, no analyses were adjusted on baseline characteristics. Within the ET group, paired baseline and follow-up measurements were compared using paired *t* test for continuous variables and Fisher’s exact test for categorical variables. Change in aerobic fitness, cardiac function, change in HR during a 10-min stand, and symptom improvement were compared between the ET and SOC arms using an analysis of covariance (ANCOVA) model with baseline as covariate for each outcome. Change in continuous measures were presented as observed mean difference ± standard deviation (SD) for within -roup paired analysis and as least-square mean difference with 95% confidence interval [LSM (95% CI)] for group comparison ANCOVA. In analysis of functional ability score, ordinal change in 10% increments and a binary indicator of any improvement vs. stable/worsening were analyzed using Fisher’s exact test. Exploratory analysis of the relationship between activity load and meaningful change in VO_2_ peak (2.8 mL/min/kg or greater) was conducted using linear regression and logistic regression analyses. Study data were collected and managed using REDCap electronic data capture tools hosted at Mayo Clinic [19, 20].

## Results

**Fig. 1:**
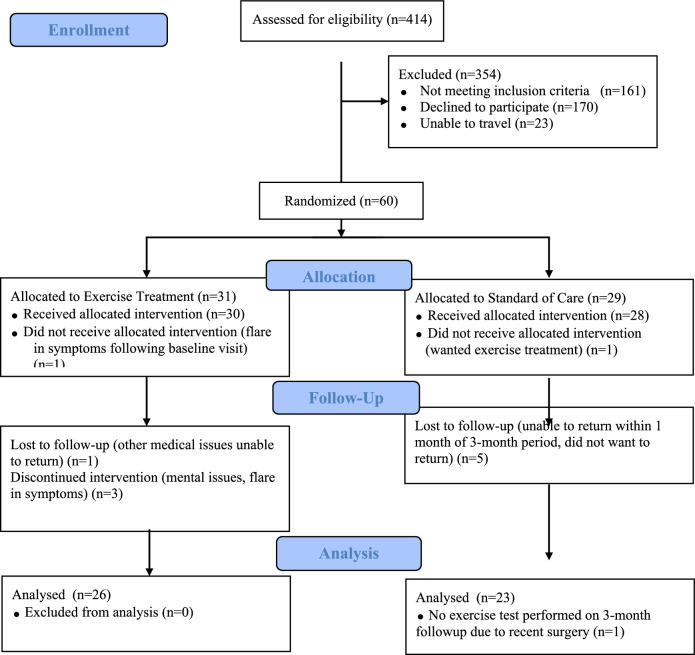
CONSORT diagram

Sixty subjects were recruited and completed the baseline visit. Eleven participants withdrew from the study. Five individuals in the ET group did not complete all the training and follow-up visits because of illness, for two of which this was a flare in autonomic symptoms. Six individuals in the SOC group did not return for follow-up testing; for two subjects contact was lost, one withdrew when not in the ET group, and three individuals could not perform the follow-up visit within a month of the 3-month treatment period ending (Fig. 1). Out of 30 ET subjects only 13% stopped as a result of illness or worsening symptoms. Fourteen subjects were from out of state, with five randomized to the ET group and nine to the SOC. One out of state participants dropped out before starting training (ET group) and three were unable to return to complete the 3-month follow-up visit (SOC group). There were seven participants who trained virtually, with two being local, but who needed the virtual accommodation. Seven individuals with hEDS completed the study, with another four withdrawing. Half (3/6) of those who dropped out of the SOC group had hEDS and in the ET group 20% (1/5) of those who dropped out had hEDS. Additionally, there were five participants who had been diagnosed with hypermobility spectrum disorder who completed the study and another three in the ET group who withdrew. As such, the data reported reflects the results of the 49 subjects who completed the study. There was no significant difference between ET and SOC groups in baseline demographics characteristics (Table 1) or baseline aerobic fitness [61.5 (15.2) vs. 66.3 (15.7) % predicted, *p* = 0.2103], 24 h urine sodium [133.4 (44.4) vs. 154.9 (80.8) mmol/24 h, *p* = 0.4408], bioimpedance measured total body water [44.8 (8.1) vs. 43.6 (4.3) %, *p* = 1.000]. Description of the within ET group effects can be found in the supplemental material.

**Table 1:** Participant demographics

	Group		Total (*N* = 49)	*P* value
	Exercise treatment (*N* = 26)	Standard of care (*N* = 23)		
Dropout,* n*	5	6	11	
Age, years	33 (11)	37 (11)	35 (11)	0.2251^a^
Range	18.0, 66.0	19.0, 57.0	18.0, 66.0	
Sex, *n* (%)				0.6119^b^
Female	23 (88.5%)	22 (95.7%)	45 (91.8%)	
Male	3 (11.5%)	1 (4.3%)	4 (8.2%)	
Dropout	5F	6F	11F	
Height (cm)	170.3 (8.8)	167.7 (6.4)	169.1 (7.8)	0.3822^a^
Weight (kg)	73.5 (15.7)	80.6 (20.7)	76.8 (18.4)	0.2662^a^
Race/ethnicity, *n* (%)				0.0961^b^
White, non-Hispanic	26 (100.0%)	21 (87.5%)	47 (94.0%)	
Hispanic/Latino (H/L)	0 (0%)	2 (8.7%)	2 (4.1%)	
Dropout, *n*	3W, 1 H/L	6W, 1 H/L	9W, 2 H/L	
Hypermobile Ehlers–Danlos syndrome, *n* (%)	4 (17.4%)	3 (13.0%)	7 (12.2%)	1.0000^b^
Dropout, *n*	1	3	4	
Hypermobility spectrum disorder, *n* (%)	2 (7.7%)	3 (12.5%)	5 (6.1%)	0.6550^b^
Dropout, *n*	3	0	3	
Medications				
Beta blocker, *n* (%)	9 (34.6%)	10 (43.4%)	19 (38.8%)	0.5688^b^
Alpha agonist, *n* (%)	3 (11.5%)	5 (21.7%)	8 (16.3%)	0.4481^b^
Cromolyn, *n* (%)	4 (15.4%)	2 (8.7%)	6 (12.2%)	0.6707^b^
Ivabradine, *n* (%)	2 (7.7%)	3 (13.0%)	5 (10.2%)	0.6550^b^
None, *n* (%)	11 (42.3%)	6 (26.1%)	17 (34.7%)	0.3675^b^
Comorbidities				
Migraine or headaches, *n* (%)	13 (50.0%)	10 (43.5%)	23 (46.9%)	0.7762^b^
MCAS, *n* (%)	6 (23.1%)	5 (21.7%)	11 (22.4%)	1.0000^b^
Asthma, *n* (%)	3 (11.5%)	2 (8.7%)	5 (10.2%)	1.0000^b^
Fatigue, *n* (%)	3 (11.5%)	2 (8.7%)	5 (10.2%)	1.0000^b^
Pain, *n* (%)	2 (7.7%)	3 (13.0%)	5 10.2%)	0.6550^b^
IBS, *n* (%)	1 (3.8%)	3 (13.0%)	4 (8.2%)	0.3297^b^
Anemia, *n* (%)	2 (7.7%)	1 (4.3%)	3 (6.1%)	1.0000^b^
Sjogrens, *n* (%)	1 (3.8%)	2 (8.7%)	3 (6.1%)	0.5943^b^

Values are mean (SD)

^a^Kruskal–Wallis *p* value

^b^Fisher’s exact *p* value

### Aerobic fitness and exercise tolerance

**Fig. 2:**
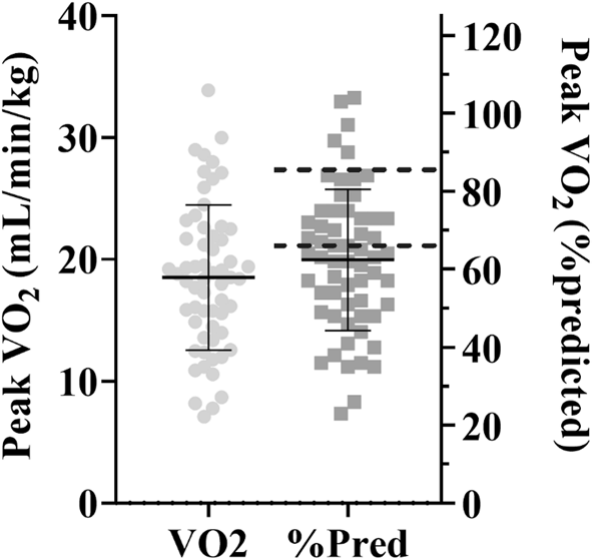
Baseline maximal oxygen consumption (VO_2_ peak) of all 60 participants recruited. With absolute VO_2_ peak on the left (gray circles) and relative VO_2_ peak as a percentage of predicted on the right (dark gray squares). Hatched lines designate level of deconditioning with mild classified as VO_2_ peak 65–85% and severe classified as < 65%

**Fig. 3:**
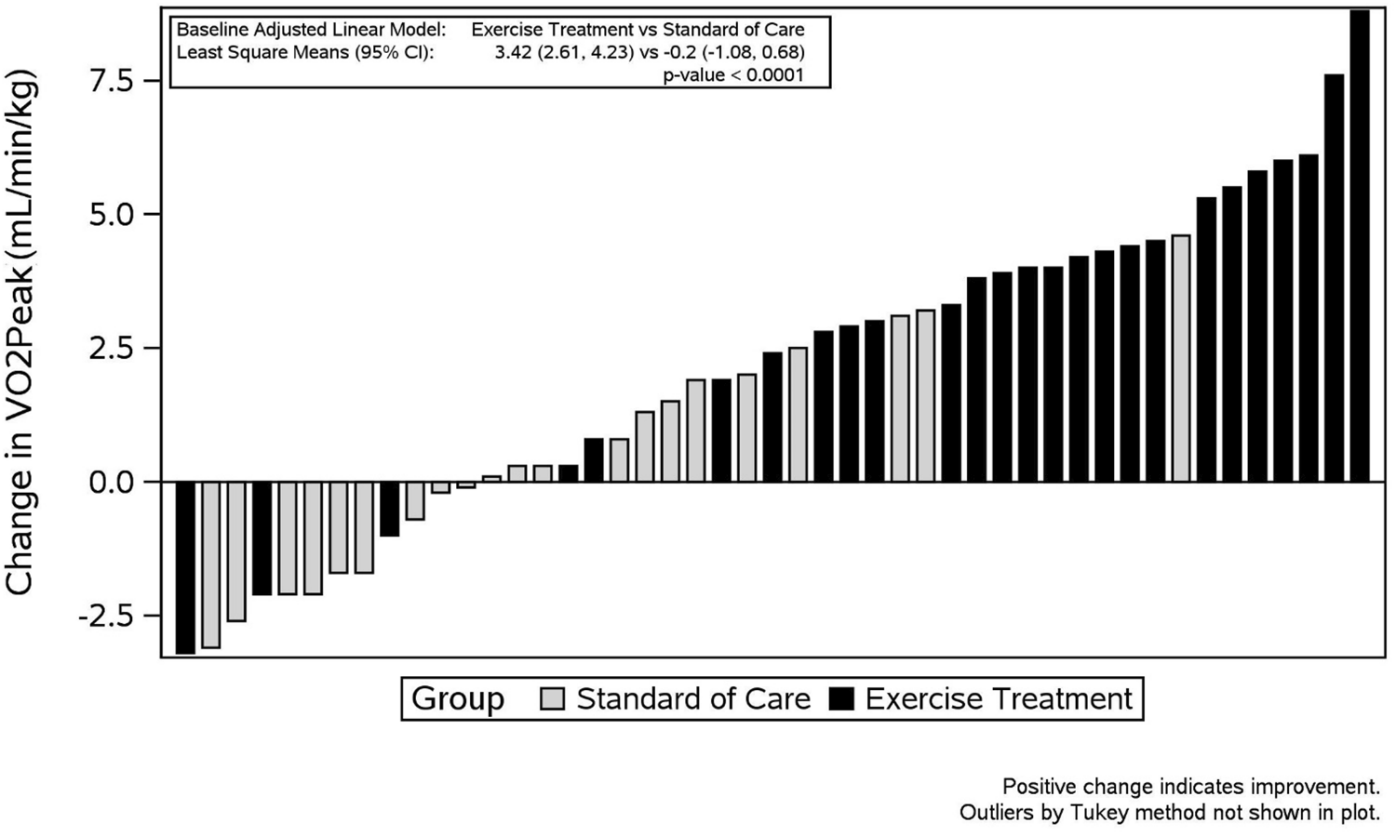
Change in aerobic fitness (peak oxygen consumption (VO_2PEAK_) in mL/min/kg) from baseline to 3 months post for each participant with those in the ET group (black) and those in the SOC group (gray). Average change in VO_2PEAK_ [3.42 (2.61, 4.23) vs. − 0.2 (− 1.08, 0.68), *p* < 0.0001], least-square means (95% CI), ET vs. SOC respectively

Peak oxygen consumption (VO_2PEAK_) is the maximal rate of oxygen consumption measured during exercise and provides an assessment of aerobic fitness. Out of all subjects recruited 55/60 or 92% were deconditioned (VO_2PEAK_ < 85% predicted) and 33/60 or 55% were severely deconditioned (VO_2PEAK_ < 65% predicted) at baseline (Fig. 2). The improvement in aerobic fitness from baseline to 3 months post was significantly higher in the ET group than in the SOC group (VO_2PEAK_ 3.4 vs. − 0.2 mL/min/kg, or 11.8% vs. − 1.2% predicted, ET vs. SOC respectively, *p* < 0.0001, Fig. 3). The change in cardiac output or stroke volume at submaximal exercise (25 W matched workload and anaerobic threshold) between the baseline and 3-month post visit was not different between groups (Table 2). Exercise training resulted in a lower heart rate at the anaerobic threshold workload, which was not observed in the SOC group (*p* = 0.0142). There was a slight drop in total peripheral resistance at both submaximal workloads which was similar between groups (*p* = 0.9820). There was a significantly larger change in peak O_2_ pulse in the ET group compared to SOC, both absolute (*p* = 0.0051) and predicted (*p* = 0.0023, Table 2).

**Table 2:** Change in markers of exercise tolerance/efficiency at submaximal workloads

	Exercise treatment	Standard of care	*P* value
Matched (25 W)			
VO_2_ (mL/min/kg)	− 0.4 (− 0.8, 0.1)	− 0.53 (− 1.0, − 0.1)	0.5915
Heart rate (bpm)	4.4 (− 9.2, 0.4)	− 3.6 (− 8.8, 1.7)	0.8200
Cardiac output (L/min)	− 0.02 (− 0.55, 0.51)	− 0.19 (− 0.69, 0.31)	0.6459
Stroke volume (mL)	1.34 (− 3.71, 6.38)	0.7 (− 4.07, 5.47)	0.8540
Total peripheral resistance (Woods units)	− 1.22 (− 3.7, 1.27)	0.58 (− 1.9, 3.06)	0.3091
RPE	− 1.3 (− 1.9, -0.7)	− 1.0 (− 1.6, 0.4)	0.4482
Dyspnea	− 0.8 (− 1.1, − 0.4)	− 0.4 (− 0.8, 0.0)	0.1475
Anaerobic threshold			
VO_2_ (mL/min/kg)	− 0.03 (− 0.56, 0.61)	− 0.29 (− 0.39, − 0.98)	0.5553
Heart rate (bpm)	− 8.7 (− 13.9, − 3.4)	1.6 (− 4.5, 7.8)	0.0142
Cardiac output (L/min)	− 0.04 (− 0.6, 0.51)	0.01 (− 0.56, 0.58)	0.8942
Stroke volume (mL)	3.32 (− 1.33, 7.97)	0.94 (− 3.86, 5.73)	0.4729
Total peripheral resistance (Woods units)	− 0.81 (− 1.82, 0.2)	− 0.79 (− 1.87, 0.28)	0.9820
RPE	− 2.2 (− 3.2, − 1.3)	− 0.6 (− 1.7, 0.6)	0.0287
Dyspnea	− 1.4 (− 2.0, − 0.8)	− 0.3 (− 1.0, 0.4)	0.0229
Peak			
Peak O_2_ pulse	1.24 (0.72, 1.76)	0.11 (− 0.45, 0.67)	0.0051
Peak O_2_ pulse (% predicted)	9.74 (5.74, 13.73)	0.22 (− 4.12, 4.57)	0.0023
Mean arterial pressure (mmHg)	− 2.49 (− 5.77, 0.78)	− 5.68 (− 9.24, -2.11)	0.1946

Least mean square difference (95% CI)

*VO*_*2*_ oxygen consumption, *RPE* Rating of Perceived Exertion

**Fig. 4:**
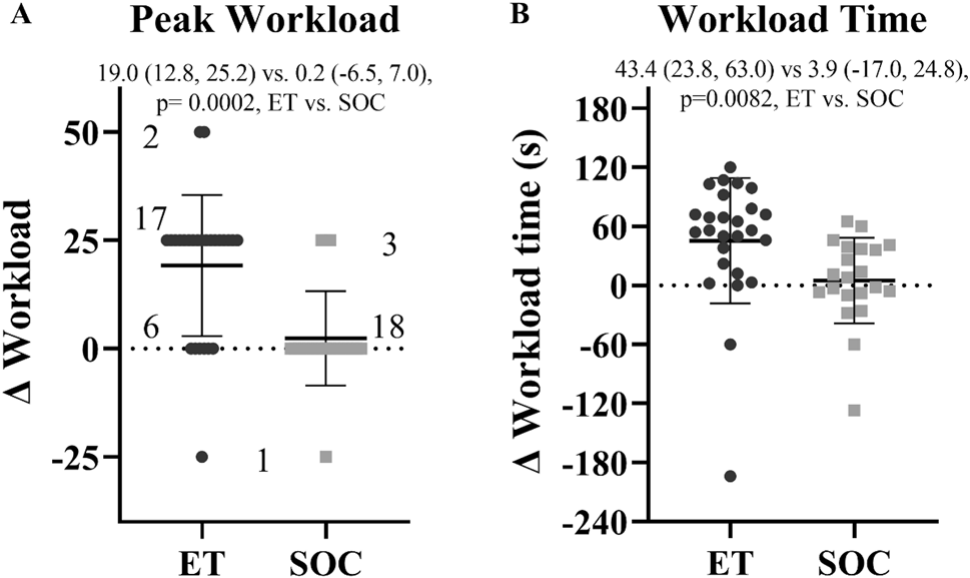
**A** Change in peak workload in watts [19.0 (12.8, 25.2) vs. 0.2 (− 6.5, 7.0), *p* = 0.0002]. **B** Change in total time in seconds spent in final workload [43.4 (23.8, 63.0) vs. 3.9 (− 17.0, 24.8), *p* = 0.0082]. Individuals in the ET group (black circles) and those in the SOC group (gray squares). Least-square means (95% CI), ET vs. SOC respectively

In review of improvements in exercise tolerance at the submaximal workloads, no significant difference was observed at the matched 25-W workload, but those in the exercise group demonstrated a greater reduction in heart rate, dyspnea rating, and RPE at the anaerobic threshold workload compared to those in the SOC group (Table 2). Additionally, a greater number of individuals in the exercise group demonstrated a delay in symptom worsening or onset based on a shift to lower reported Buffalo Concussion Treadmill Test (BCTT) symptom scores/rating at the matched (*p* = 0.0373) and anaerobic threshold workloads (*p* = 0.0149, Table 3). With this improved exercise tolerance, peak workload increased by 19 W on average or one stage for those in the ET group compared to no change on average for those in the SOC group (*p* = 0.0002, Fig. 4). The highest workload achieved was also tolerated for longer on average for those in the ET group lasting almost a minute longer in the final stage compared to those in the SOC who only tolerated several seconds of the next stage (*p* = 0.017, Fig. 4).

**Table 3:** Change in symptom onset or worsening during exercise

Symptoms during submaximal exercise	Exercise treatment	Standard of care	*P* value
Change in BCTT score (matched 25 W)	*n* (%)	*n* (%)	0.0373
− 3	0 (0.0%)	1 (4.8%)	
− 2	2 (8.3%)	0 (0.0%)	
− 1	13 (54.2%)	5 (23.8%)	
0	7 (29.2%)	11 (52.4%)	
1	1 (4.2%)	4 (19.0%)	
2	1 (4.2%)	0 (0.0%)	
Change in BCTT score (anerobic threshold)	*n* (%)	*n* (%)	0.0149
− 3	2 (8.3%)	0 (0.0%)	
− 2	2 (8.3%)	0 (0.0%)	
− 1	12 (50.0%)	3 (16.7%)	
0	6 (25.0%)	7 (38.9%)	
1	2 (8.3%)	6 (33.3%)	
2	0 (0.0%)	2 (11.1%)	

Fisher exact *p* value

*BCTT* Buffalo Concussion Treadmill Test symptom severity

### Symptom improvement (COMPASS 31, SF-36, and FAS)

Individuals in the ET group reported greater symptom improvement in the COMPASS 31 assessment domains of orthostatic intolerance (*p* = 0.0219) and vasomotor (color changes in skin) (*p* = 0.037, Table 4). The degree of improvement in the total COMPASS 31 symptom severity score was not significantly different between the ET and SOC groups (*p* = 0.0925). Two ET participants reported no longer having orthostatic symptoms after the 3 months of training.

**Table 4:** Change in COMPASS 31 subscale scores

	Exercise treatment	Standard of care	*P* value
Orthostatic intolerance	− 1.98 (− 2.65, − 1.3)	− 0.81 (− 1.53, -0.09)	0.0219
Vasomotor	− 0.28 (− 0.53, − 0.04)	0.1 (− 0.16, 0.36)	0.0370
GI	− 2.62 (− 4.13, − 1.12)	− 1.91 (− 3.51, − 0.31)	0.5170
Secretomotor	− 0.83 (− 1.33, − 0.33)	− 0.37 (− 0.9, 0.16)	0.2162
Bladder	− 0.34 (− 0.79, 0.11)	− 0.27 (− 0.75, 0.21)	0.8234
Pupilomotor	− 1.48 (− 2.5, − 0.46)	− 0.63 (− 1.72, 0.46)	0.2605
Total autonomic symptom score	− 11.38 (− 15.38, − 7.38)	− 6.49 (− 10.58, − 2.4)	0.0925

Least mean square difference (95% CI)

**Fig. 5:**
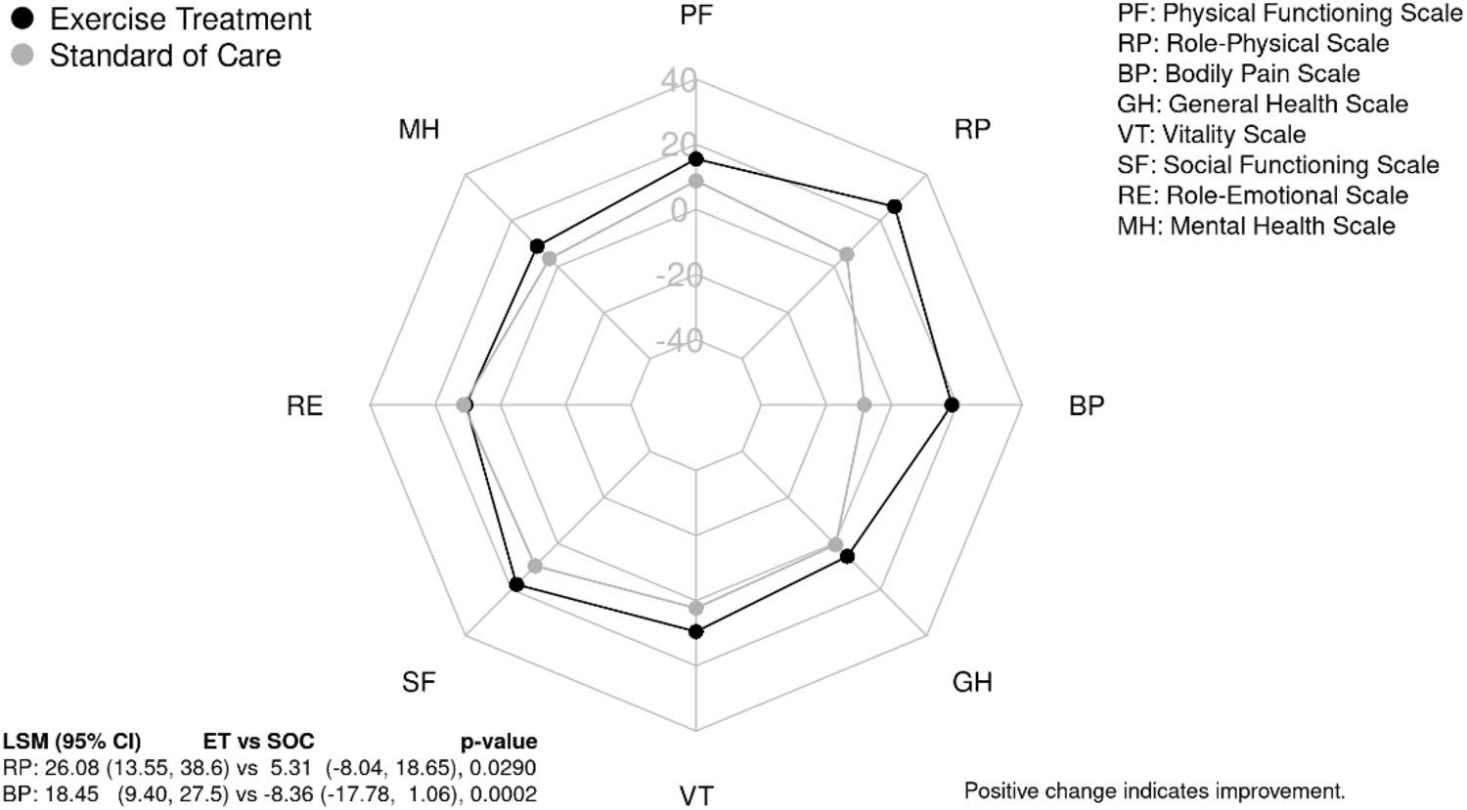
Change in SF-36 domains from baseline to 3 months post. RP, role-physical [26.08 (13.55, 38.6) vs. 5.31, (− 8.04, 18.65), *p* = 0.0290]; BP, bodily pain [18.45 (9.4, 27.5) vs. − 8.36 (− 17.78, 1.06, *p* = 0.0002]. All other domains: *PF* physical functioning, *GH* general health, *VT* vitality, *SF* social functioning, *RE* role-emotional, *MH* mental health, *p* > 0.05. For those in the ET group (black) and those in the SOC group (gray) with positive change indicating improvement. Least-square means (95% CI), ET vs. SOC respectively

In the SF-36 assessment, the ET group showed a significant improvement in bodily pain whereas the SOC showed a worsening on average (*p* = 0.0002, Fig. 5). The role-physical scale or the degree to which one’s physical health is affecting work or other activities showed greater improvement for those in the ET group (*p* = 0.0290, Fig. 5). The ET group reported a statistically significantly improvement in the physical component summary score as compared to the SOC group [7.03 (3.9, 10.16) vs. 0.4 (− 2.93, 3.73), *p* = 0.0055]. There was no significant difference in the change in the SF-36 mental component summary scores between groups [3.66 (0.02, 7.31) vs. 3.07 (− 0.81, 6.94), *p* = 0.8231].

There was no significant difference in the frequency of positive change in the reported functional ability in 10% increments, nor in number of individuals who reported any improvement vs. worsening or staying stable in their functional ability score (Table 5). However, functional ability did appear to be shifting in the positive direction for those in the ET group. Following the exercise training 13 individuals reported their functional ability as 80% or greater vs. only seven individuals at baseline which means these individuals felt they were able to work at least part-time. Additionally, at baseline 12 individuals reported being at or below 50% functional ability meaning they had at best more bad days than good, which reduced to nine individuals after the 3 months of exercise.

**Table 5:** Change in reported functional ability score

	Group		
	Exercise treatment	Standard of care	*P* value
Functional ability score change (%)	*n* (%)	*n* (%)	0.35
− 20	2 (7.7%)	2 (9.1%)	
− 10	1 (3.8%)	3 (13.6%)	
0	11 (42.3%)	7 (31.8%)	
10	3 (11.5%)	4 (18.2%)	
20	4 (15.4%)	0 (0.0%)	
30	1 (3.8%)	3 (13.6%)	
40	4 (15.4%)	2 (9.1%)	
60	0 (0.0%)	1 (4.5%)	
Change in FAS	*n* (%)	*n* (%)	1.00
Worsening or stable FAS	14 (53.8%)	12 (54.5%)	
Any Improvement in FAS	12 (46.2%)	10 (45.5%)	

Fisher exact *p* value

*FAS* functional ability score

### Orthostatic tolerance 10 min stand test

**Fig. 6:**
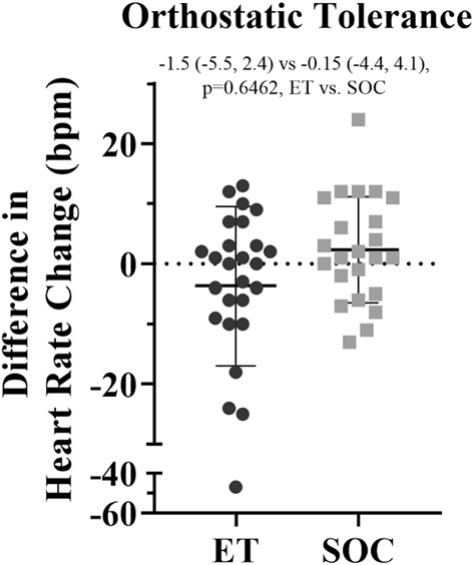
Difference in change in heart rate with standing between the baseline and 3 month post 10 min stand test [− 1.5 (− 5.5, 2.4) vs. -0.15 (− 4.4, 4.1), *p* = 0.6462] for those in the ET group (black circles) and those in the SOC group (gray squares). Least-square means (95% CI), ET vs. SOC respectively

In the tolerance of positional changes assessment using the 10 min stand test, those in the exercise group demonstrated a 4-bpm increase on average in their resting supine heart rate (77 ± 15 vs. 81 ± 13 bpm, baseline vs. 3 months post, *p* = 0.0022). The difference in the change in heart rate with standing was not significantly different between the ET and SOC groups (*p* = 0.6462) (Fig. 6).

### Exercise dose

Comparing the exercise habits of participants over the 3-month treatment period, individuals in the ET group were more likely to complete at least 2 sessions/week, the study recommended 3 sessions/week and had fewer weeks with no exercise sessions. During their exercise sessions average heart rate during the session was higher in those in the ET group. The percentage of participants training in the suggested target heart rate zone was greater in the ET group. As a result, the average weekly aerobic activity load was significantly higher in the ET group (Table 6). Average weekly activity load as a continuous measure was not significantly predictive of the change in VO_2_ peak (*p* = 0.054), but on the basis of the linear regression model for every 10-unit change in average weekly activity load there is a 0.32 mL/min/kg increase in VO_2_ peak. Having an average weekly activity load of 32 or greater increased the change in VO_2_ peak by 3.04 mL/min/kg on average [95% CI (0.90, 5.19), *p* = 0.0064]. In predicting a change in VO_2_ peak of 2.8 mL/min/kg or greater, those who had an average weekly activity load of 32 or greater were 7.33 times more likely to see an improvement [OR (95% CI) 7.33 (1.41, 38.12),* p* = 0.0179].

**Table 6:** Metrics of exercise dose over the 12-week intervention

	Exercise treatment	Standard of care	*P* value
Exercise sessions/week over 12 weeks			
Weeks with zero sessions/week	2 ± 2	5 ± 5	0.0202^b^
Weeks with ≥ 2 sessions/week	8 ± 3	5 ± 4	0.0040^b^
Weeks with recommended 3 sessions/week	4 ± 3	2 ± 2	0.0083^b^
Training session average HR (bpm)	122 ± 13	114 ± 12	0.0288^b^
Training in target HR zone *n* (% of participants)	19/26 (73.1%)	6/17 (35.3%)^a^	0.0259^c^
Average weekly aerobic activity load	61.1 ± 27.1	38.3 ± 32.1	0.0144^b^

Values mean ± SD

^a^Six participants in the standard of care group logged no sessions during 12-week intervention

^b^Kruskal–Wallis *p* value

^c^Fisher’s exact *p* value

## Discussion

Data from this study demonstrated that providing minimal supervision resulted in better improvements in exercise tolerance and symptoms than receiving exercise recommendation or guidance with no supervision. Additionally, data suggested that improved efficacy is likely due to increased likelihood of exercise being performed at the correct dose (frequency, duration, and intensity).

In line with previous work, the individuals with POTS recruited in this study were primarily deconditioned and over half were severely deconditioned. Although symptoms for individuals with POTS are caused by a variety of factors, results of this study highlight that an exercise intervention can improve deconditioning with an observed increase in peak VO_2_ of 3.4 mL/min/kg on average, which was almost 12% predicted and meant a shift from being severely deconditioned to moderately deconditioned for the ET group. This level of improvement was as good if not better (26.1 to 28.9 mL/min/kg pre to post training) than has been previously demonstrated by Shibata et al. in a 3-month supervised training program in prior studies [9].

One of the limitations of a prior community-based POTS registry study was that it had a 59% dropout rate, and within those dropouts 40% of participants stopped because training was too difficult (59/148 enrolled) and seven continued training, but at a lower intensity [10]. In the current study, out of 30 ET subjects only 16% stopped because of illness or worsening symptoms. Additionally, the current study enrolled participants with hEDS, a group which was excluded in the prior studies. Although the aerobic and strength training recommendations were quite similar between the two studies, one difference was that the target heart rate zone provided to participants was 70–75% predicted max heart rate. Because many of these patients can be taking medication that slows or blunts heart rate response, using predicted values can make the suggested training zone very impersonal and possibly not attainable or too difficult. In contrast, in the current study peak heart rate and heart rate response to exercise were measured not estimated, and participants were instructed to utilize breathing and symptoms first and heart rate as a secondary means to confirm they were challenging themselves. The results of this study suggest that the current methods of exercise prescription, delivery, and guidance used in this study appear to be more effective for a wider range of individuals with POTS.

Although the current study did not have a complete resolution of symptoms which 71% of participants reported in the POTS registry community-based study, those in the ET group demonstrated greater improvement in orthostatic symptoms and a greater improvement in bodily pain (SF-36) compared to the SOC group. Although the 3-month exercise training program may not have resulted in a significant change in reported functional ability score or the orthostatic tachycardia with standing, these were trending in the positive direction. With the significantly greater improvement in exercise tolerance based on improvements in peak workload, time in peak workload, and delay in symptom worsening or onset, as well as signs of improved efficiency at submaximal workloads, this suggests better tolerance of activities of daily living. Further, it has recently been proposed that exercise can provide beneficial vascular conditioning [21] and work by Hambrecht et al. demonstrated that aerobic exercise training improved endothelium-dependent dilation in patients with coronary artery disease [22]. Chopoorian et al. demonstrated that individuals with POTS have reduced flow-mediated dilation [23] and as such collectively the data suggests that continued effective and progressive exercise training could elicit improvements in aerobic capacity and improvement in endothelial function in individuals with POTS. For some participants a longer intervention period may have been needed to see significant improvement in functional ability score and orthostatic tachycardia, as it is known that there is variability in the individual response to an exercise intervention and everyone was starting at a different baseline fitness and severity of disease, but everyone is trainable and one hypothesizes that with continued personalized progression of the exercise dose all will see a significant improvement [24–26].

During the exercise test cardiac output was measured at rest, matched workload of 25 W, and anaerobic threshold to assess improvements in cardiac function with the semi-supervised exercise training anticipating both a reduction in tachycardia and improvement in pumping efficiency or stroke volume. Similar to previous work those in the ET group demonstrated a reduction in heart rate at all submaximal workloads and an increase in stroke volume at anaerobic threshold which maintained cardiac output within the ET group [9]. However, the degree of change between visits was not significantly different from what was observed in the SOC group. The improvement in peak O_2_ pulse, which is the indirect surrogate for stroke volume, was significantly different between groups; this would be driven by the significant improvement in VO_2_ and peak heart rate which determine O_2_ pulse. The lack of difference in the change in stroke volume between groups is possibly driven by the nature of the timing of the cardiac output rebreathe measurement as the matched and anaerobic threshold workloads were kept the same between visits. In the setting of an improvement in fitness which occurred for the majority of ET group participants these resistance/workloads where the cardiac output measurement took place may not have elicited a sufficient increase in demand to necessitate a large increase in stroke volume due to improvements in efficiency. In support of this hypothesis, VO_2_ was lower at the matched workload and heart rate was lower at anaerobic threshold within those in the ET group.

To achieve the level of improvement observed in this study, the data suggests that exercise dose needs to be sufficient. Work by Thomas et al. demonstrated that only 34% of patients with diabetes start a physical activity routine when provided as a recommended treatment for their condition, and of these that start to exercise only 9% of these patients perform the exercise at a sufficient intensity to be effective [27]. In agreement with this observation, this current study found that those in the SOC group who were exercising were typically not performing enough sessions/week or at the proper intensity and as a result did not see the desired improvements in aerobic fitness or symptoms. Improvement in VO_2PEAK_ or aerobic or cardiorespiratory fitness is a key metric of an effective exercise program. Peak oxygen consumption was recently demonstrated to be one of the best prognostic markers for mortality independent of age, sex, and race [28]. Further for every 3.5 mL/min/kg improvement this provides an estimated 16% reduction in mortality in persons with and without cardiovascular disease [29, 30]. Although not the primary objective, the study identified a threshold or cut point of having an average weekly activity load of 32 or greater which was predictive of improvement in VO_2PEAK_. This would equate to accumulating 42 min/week where average heart rate was at 75% of an individual’s maximum heart rate (HRmax), 27 min/week where average heart rate was at 85% HRmax, or 64 min/week where average heart rate was 50% HR max.

Aspects of the implemented semi-supervised ET program utilized in this study are important to highlight for those trying to implement a similar program. First, cardiopulmonary exercise testing followed by a face-to-face consultation with a clinical understanding of condition-specific limitation allowed for a personalized exercise prescription for each participant. At a minimum, it would be beneficial to have all participants complete a staged progressive exercise test/session with heart rate and symptom monitoring while on their normal medication in the gym where training is occurring or as part of the initial exercise consultation if a clinical cardiopulmonary exercise test is not feasible. Second, receiving occasional real-time instruction in a group environment allows participants to gain comfort in knowing how best to train, can ease fears or anxiety about exercising with their health condition or conditions, and provides guidance on when to progress based on how the prior week’s sessions went. This bridges the gap between the exercise consultation and independent training in a more administratively feasible option. Third, the ability to attend supervised sessions virtually or in-person made the program accessible to those who were from out of state and was more convenient for busy working individuals regardless of location. Fourth, prior work evaluating the characteristics of physical activity promotion programs which are effective for causing and maintaining a behavioral change identified that self-monitoring and social connectivity were two features that appeared to improve initiation rates and maintenance of behavior/lifestyle modifications [31, 32]. This study utilized group training environments for in-person and virtual sessions which provided social support from others who have similar symptoms/conditions, but may have been at a different point in their training. Moreover, mobile application use for tracking exercise sessions to provide accountability and helped bridge the gap between supervised exercise training to the semi-supervised model. Collectively, the proposed evaluation of semi-supervised remote self-training has potential to improve patient care by providing an effective and affordable treatment option, and similar programs have been shown to be successful in other populations [33–37].

## Study limitations

Although this study added to the body of literature by evaluating the effects of an exercise program in comparison to the SOC in a randomized control trial and included those with hEDS, there were limitations, which require further research to answer. The weekly activity load was self-reported and dependent on participants tracking each session in the app, rather than using an activity monitor which would collect activity data continuously. The app with chest strap heart rate monitor was chosen to allow for heart rate monitoring in real time during training sessions, but does present a limitation as the tracked sessions may underrepresent total activity of participants as formal continuous aerobic exercise sessions may have been performed but never tracked. Future work should utilize an accelerometer or an activity monitor which could monitor changes in all activity not just tracked activity habits. Although the study was powered and designed to evaluate improvement in aerobic fitness, improvement in symptoms, quality of life, and functional ability is most valuable for clinicians. The questionnaires used in this study did try to assess this, but with the functional ability score, participants had to pick a discrete percentage according to the description that best described their current state; many found this difficult as they may have improved but did not meet all the requirements described in the next category (i.e., ability to start working or go to school part-time or full-time). As such, this may have limited the sensitivity to detect small, but meaningful improvements in functional ability and a longer intervention period may have been needed. The mechanism for improvement in aerobic fitness and endothelial function has been previously demonstrated, highlighting that additional research is needed to evaluate longer exercise intervention periods and should also include evaluation of endothelial function. Finally, with over 50% of the study population having headaches or migraines and this being a common comorbidity in this population, it would have been advantageous to evaluate the effect of the ET on severity or frequency of headaches/migraines.

## Conclusion

Providing minimal supervision resulted in better improvements in exercise tolerance and symptoms than receiving an exercise recommendation or guidance with no supervision. Improved efficacy is likely due to increased likelihood of exercise being performed at the correct dose. Healthcare systems can better meet the needs and improve the health of numerous patients by bridging the gap and providing a guided exercise prescription with potentially a better understanding of condition-specific limitations than most trainers. The semi-supervised exercise training model has the potential to improve patient care by providing an effective exercise prescription that is clinically possible as an affordable treatment either delivered in-person or virtually. This can provide more patients with an opportunity to take the first step, give them the comfort of supervision as they are getting started, and help keep them accountable until they get into a routine, and make exercise a habit that they can then continue on their own.

### Supplementary Information

Below is the link to the electronic supplementary material.

Supplementary file1 (DOCX 1025 kb)

## Data Availability

Not applicable.

## Abbreviations

VO_2PEAK_: Peak oxygen consumption
COMPASS 31: Composite Autonomic Symptom Score
ET: Exercise training
FAS: Functional ability score
hEDS: Hypermobile Ehlers–Danlos syndrome
POTS: Postural orthostatic tachycardia syndrome
RPE: Rating of perceived exertion
SF-36: Short Form 36 Health Questionnaire
SOC: Standard of care
VO_2_: Oxygen consumption

## References

[1] Fu Q, Levine BD (2015). Exercise in the postural orthostatic tachycardia syndrome. Auton Neurosci.

[2] Low PA, Opfer-Gehrking TL, Textor SC, Benarroch EE, Shen WK, Schondorf R (1995). Postural tachycardia syndrome (POTS). Neurology.

[3] Low PA, Sandroni P, Joyner M, Shen WK (2009). Postural tachycardia syndrome (POTS). J Cardiovasc Electrophysiol.

[4] Sandroni P, Opfer-Gehrking TL, McPhee BR, Low PA (1999). Postural tachycardia syndrome: clinical features and follow-up study. Mayo Clin Proc.

[5] Parsaik A, Allison TG, Singer W, Sletten DM, Joyner MJ, Benarroch EE (2012). Deconditioning in patients with orthostatic intolerance. Neurology.

[6] Fu Q, Vangundy TB, Galbreath MM, Shibata S, Jain M, Hastings JL (2010). Cardiac origins of the postural orthostatic tachycardia syndrome. J Am Coll Cardiol.

[7] Saltin B, Blomqvist G, Mitchell JH, Johnson RL, Wildenthal K, Chapman CB (1968). Response to exercise after bed rest and after training. Circulation.

[8] Masuki S, Eisenach JH, Schrage WG, Johnson CP, Dietz NM, Wilkins BW (2007). Reduced stroke volume during exercise in postural tachycardia syndrome. J Appl Physiol.

[9] Shibata S, Fu Q, Bivens TB, Hastings JL, Wang W, Levine BD (2012). Short-term exercise training improves the cardiovascular response to exercise in the postural orthostatic tachycardia syndrome. J Physiol.

[10] George SA, Bivens TB, Howden EJ, Saleem Y, Galbreath MM, Hendrickson D (2016). The international POTS registry: evaluating the efficacy of an exercise training intervention in a community setting. Heart Rhythm.

[11] American College of Sports Medicine. Exercise is medicine. https://www.exerciseismedicine.org/ (2007). Accessed Sept 2021.

[12] Schofield JR, Chemali KR (2019). Intravenous immunoglobulin therapy in refractory autoimmune dysautonomias: a retrospective analysis of 38 patients. Am J Ther.

[13] Borg E, Borg G, Larsson K, Letzter M, Sundblad BM (2010). An index for breathlessness and leg fatigue. Scand J Med Sci Sports.

[14] Leddy JJ, Willer B (2013). Use of graded exercise testing in concussion and return-to-activity management. Curr Sports Med Rep.

[15] Hsia CC, Herazo LF, Ramanathan M, Johnson RL (1995). Cardiac output during exercise measured by acetylene rebreathing, thermodilution, and Fick techniques. J Appl Physiol.

[16] Snyder EM, Johnson BD, Beck KC (2005). An open-circuit method for determining lung diffusing capacity during exercise: comparison to rebreathe. J Appl Physiol.

[17] Wheatley CM, Baldi JC, Cassuto NA, Foxx-Lupo WT, Snyder EM (2011). Glycemic control influences lung membrane diffusion and oxygen saturation in exercise-trained subjects with type 1 diabetes: alveolar-capillary membrane conductance in type 1 diabetes. Eur J Appl Physiol.

[18] Wheatley CM, Morgan WJ, Cassuto NA, Foxx-Lupo WT, Daines CL, Morgan MA (2013). Exhaled breath condensate detects baseline reductions in chloride and increases in response to albuterol in cystic fibrosis patients. Clin Med Insights Circ Respir Pulm Med.

[19] Harris PA, Taylor R, Thielke R, Payne J, Gonzalez N, Conde JG (2009). Research electronic data capture (REDCap)–a metadata-driven methodology and workflow process for providing translational research informatics support. J Biomed Inform.

[20] Harris PA, Taylor R, Minor BL, Elliott V, Fernandez M, O'Neal L (2019). The REDCap consortium: building an international community of software platform partners. J Biomed Inform.

[21] Green DJ, O’Driscoll G, Joyner MJ, Cable NT (1985). Exercise and cardiovascular risk reduction: time to update the rationale for exercise?. J Appl Physiol (2008).

[22] Hambrecht R, Adams V, Erbs S, Linke A, Krankel N, Shu Y (2003). Regular physical activity improves endothelial function in patients with coronary artery disease by increasing phosphorylation of endothelial nitric oxide synthase. Circulation.

[23] Chopoorian AH, Wahba A, Celedonio J, Nwazue V, Smith EC, Garland EM (2021). Impaired endothelial function in patients with postural tachycardia syndrome. Hypertension.

[24] Whipple MO, Schorr EN, Talley KMC, Lindquist R, Bronas UG, Treat-Jacobson D (2018). Variability in individual response to aerobic exercise interventions among older adults. J Aging Phys Act.

[25] Tanaka H (2018). Exercise nonresponders: genetic curse, poor compliance, or improper prescription?. Exerc Sport Sci Rev.

[26] Joyner MJ, Lundby C (2018). Concepts about V O2max and trainability are context dependent. Exerc Sport Sci Rev.

[27] Thomas N, Alder E, Leese GP (2004). Barriers to physical activity in patients with diabetes. Postgrad Med J.

[28] Kokkinos P, Faselis C, Samuel IBH, Pittaras A, Doumas M, Murphy R (2022). Cardiorespiratory fitness and mortality risk across the spectra of age, race, and sex. J Am Coll Cardiol.

[29] Kodama S, Saito K, Tanaka S, Maki M, Yachi Y, Asumi M (2009). Cardiorespiratory fitness as a quantitative predictor of all-cause mortality and cardiovascular events in healthy men and women: a meta-analysis. JAMA.

[30] Boden WE, Franklin BA, Wenger NK (2013). Physical activity and structured exercise for patients with stable ischemic heart disease. JAMA.

[31] Greaves CJ, Sheppard KE, Abraham C, Hardeman W, Roden M, Evans PH (2011). Systematic review of reviews of intervention components associated with increased effectiveness in dietary and physical activity interventions. BMC Public Health.

[32] Webb TL, Joseph J, Yardley L, Michie S (2010). Using the internet to promote health behavior change: a systematic review and meta-analysis of the impact of theoretical basis, use of behavior change techniques, and mode of delivery on efficacy. J Med Internet Res.

[33] Doba N, Abe H, Hayashida N, Hinohara S (1983). Semi-supervised exercise training program for patients with coronary heart disease–its effectiveness and possible diagnostic implications for predicting their severity. Jpn Circ J.

[34] Kamata H, Ueshima K, Hashimoto K, Kobayashi N, Hiramori K (1997). Semi-supervised exercise using a step machine at home after myocardial infarction. J Cardiol.

[35] Vidoni ED, Johnson DK, Morris JK, Van Sciver A, Greer CS, Billinger SA (2015). Dose-response of aerobic exercise on cognition: a community-based pilot randomized controlled trial. PLoS One.

[36] Sigal RJ, Kenny GP, Boule NG, Wells GA, Prud'homme D, Fortier M (2007). Effects of aerobic training, resistance training, or both on glycemic control in type 2 diabetes: a randomized trial. Ann Intern Med.

[37] McBride PE, Einerson JA, Grant H, Sargent C, Underbakke G, Vitcenda M (2008). Putting the diabetes prevention program into practice: a program for weight loss and cardiovascular risk reduction for patients with metabolic syndrome or type 2 diabetes mellitus. J Nutr Health Aging.

