# Supplementary material for: Semi-supervised exercise training program more effective for individuals with postural orthostatic tachycardia syndrome in randomized controlled trial
Source: Clin Auton Res. 2023 Aug 20;33(6):659–72. doi: 10.1007/s10286-023-00970-w (PMC10751269; doi:10.1007/s10286-023-00970-w)
Supplement: Supplementary file 1 — Supplementary file1 (DOCX 1025 kb) [file 10286_2023_970_MOESM1_ESM.docx]

**Supplemental Material**

**Buffalo Concussion Treadmill Test symptom severity scale**


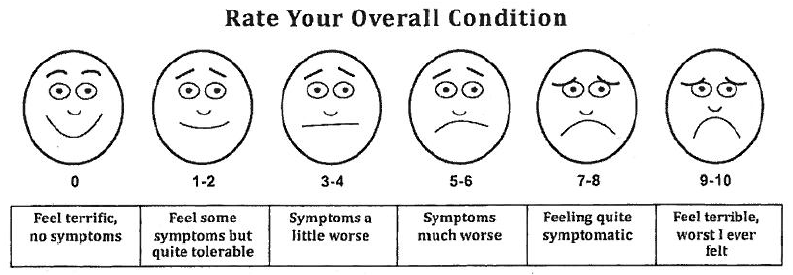


Leddy JJ, Willer B. Use of graded exercise testing in concussion and return-to-activity management. *Curr Sports Med Rep.* 2013;12(6):370-376.

**Functional Ability Score:**


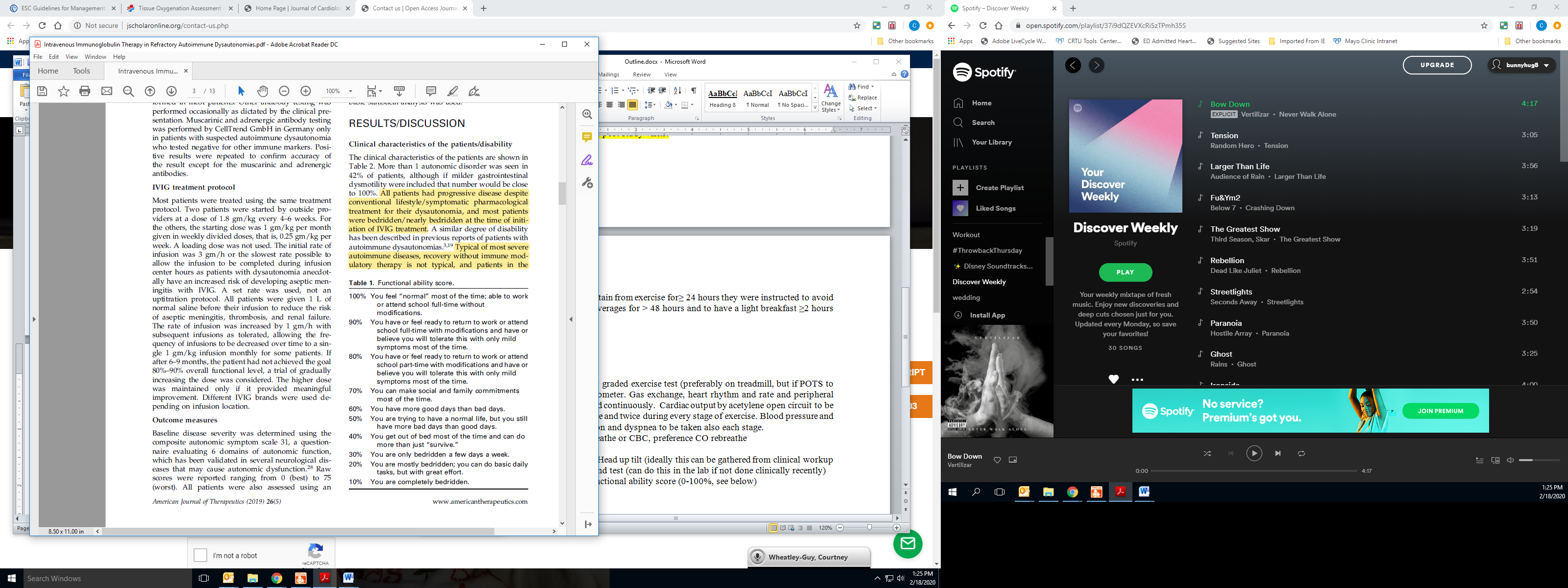
Schofield JR, Chemali KR. Intravenous Immunoglobulin Therapy in Refractory Autoimmune Dysautonomias: A Retrospective Analysis of 38 Patients. *Am J Ther.* 2019;26(5):570-582.

**Results Continued**

The following section describes the the effect of the exercise treatment on outcomes over the 12-week intervention (within ET group effect).

*Aerobic Fitness & Exercise Tolerance*

Within the ET group, there was a significant improvement in stroke volume with maintenance of cardiac output at anaerobic threshold (p<0.0001) and peak O2 pulse both absolute (p<0.0001) and predicted (p<0.0001) between visits (Table 1). Within the ET group, individuals demonstrated a significant reduction in heart rate, total peripheral resistance, RPE and dyspnea rating at both the matched and AT workload, with VO_2_ reduced only at the 25W workload (Table 1).

Table 1 Change in cardiac response and exercise tolerance within the ET group

|  | **Baseline** | **3 Months Post** | **Mean Difference (95% CI)** | **P-value** |
| --- | --- | --- | --- | --- |
| **Matched 25W** |  |  |  |  |
| **VO_2_ (mL/min/kg)** | 9.8±2.1 | 9.4±1.5 | -0.45(-0.85, -0.05) | 0.028 |
| **Heart rate (bpm)** | 113±22 | 107±16 | -6.0 (-11, -1) | 0.0132 |
| **Cardiac output (L/min)** | 6.6±1.7 | 5.9±1.1 | -0.006 (-0.38, 0.37) | 0.9747 |
| **Stroke volume (mL)** | 59.3±11.6 | 57.3±11.6 | 2.2 (-0.98, 5.4) | 0.1688 |
| **Total Peripheral Resistance Woods units)** | 15.4 ± 3.3 | 14.5 ± 2.9 | -5.1 (-7.8, 1.5) | 0.0007 |
| **RPE** | 11.0±2.5 | 9.7±2.5 | -1.3 (-1.7, 0.9) | <.0001 |
| **Dyspnea** | 2.6±1.8 | 1.7±1.4 | -0.9 (-1.2, -0.5) | <.0001 |
| **Anaerobic Threshold** |  |  |  |  |
| **VO_2_ (mL/min/kg)** | 13.8±2.8 | 13.8±3.2 | 0.04 (-0.46, 0.53) | 0.8878 |
| **Heart rate (bpm)** | 134±24 | 125±18 | -9 (-13 , -5) | <.0001 |
| **Cardiac output (L/min)** | 8.2±1.6 | 8.2±1.8 | -0.03 (-0.40, -0.34) | 0.8734 |
| **Stroke volume (mL)** | 59.6±14.3 | 64.3±14.8 | 3.5 (0.6, 6.5) | <.0001 |
| **Total Peripheral Resistance (Woods units)** | 12.6 ± 2.4 | 11.7 ± 3.1 | -8.3 (-11.1, 5.5) | <.0001 |
| **RPE** | 14.5±1.8 | 12.1±2.7 | -2.4 (-3.2, -1.6) | <.0001 |
| **Dyspnea** | 4.3±1.7 | 2.8±1.5 | 3.5 (0.6, 6.5) | <.0001 |
| **Peak** |  |  |  |  |
| **VO_2_ Peak (mL/min/kg)** | 18.8±5.0 | 22.3±5.0 | 3.4 (2.7, 4.2) | <.0001 |
| **VO_2_ Peak (% predicted)** | 62±15 | 73±17 | 11.8 (9.3,14.3) | <.0001 |
| **Peak HR (bpm)** | 164±25 | 168±20 | 5 (1,8) | 0.0293 |
| **Peak O_2_ pulse** | 8.4±2.2 | 9.7±2.2 | 1.3 (.9, 1.7) | <.0001 |
| **Peak O_2_ pulse (% predicted)** | 71±14 | 81±14 | 10 (7, 13) | <.0001 |
| **Mean arterial pressure (mmHg)** | 105.2±16.2 | 102.6±12.2 | -1.5 (-4.2, 1.2) | 0.2670 |
| RPE: rating of perceived exertion. Values mean ± SD. P<0.05 based on paired t-test | | | | |

*Symptom improvement (COMPASS 31, SF-36 and FAS)*

Within the ET group, individuals reported a significant improvement in all domains of the COMPASS 31 (Supplemental Table 2). Within the ET group, all 8 domains of health in the SF-36 showed significant improvement except role-emotional (the degree to which emotional health is affecting work or other activities) (Supplemental Table 3).

Table 2: Change in COMPASS subscale scores within the ET group

|  | **Baseline** | **3 Months Post** | **Mean Difference (95% CI)** | **P-value** |
| --- | --- | --- | --- | --- |
| **Orthostatic intolerance** | 6.2±2 | 4.4±2 | -1.8 (-2.4, -1.2) | <0.0001 |
| **Vasomotor** | 3.6±0.8 | 3.3±0.7 | -0.27 (-0.51, -0.03) | 0.0293 |
| **GI** | 10.7±5.2 | 8.2±4.9 | -2.5 (-3.8, -1.2) | 0.0003 |
| **Secretomotor** | 3.0±1.9 | 2.1±1.6 | -0.92 (-1.28, -0.57) | <0.0001 |
| **Bladder** | 1.4±1.6 | 1.1±1.5 | -0.35 (-0.59, -0.1) | 0.008 |
| **Pupilomotor** | 7.1±3.6 | 5.7±3.0 | -1.4 (-2.2, -0.5) | 0.0022 |
| **Total autonomic symptom score** | 47.7± | 37.2±12.0 | -11.0 (-14.1, -7.9) | <0.0001 |
| Values mean ± SD. P<0.05 based on paired t-test | | | | |

Table 3: Change in SF-36 domain scores within the ET group

|  | **Baseline** | **3 Months Post** | **Mean Difference (95% CI)** | **P-value** |
| --- | --- | --- | --- | --- |
| **Physical functioning** | 48.1±17.0 | 62.1±16.2 | 14.0 (9.9, 18.1) | <.0001 |
| **Role-Physical** | 4.8±14.2 | 32.7±39.2 | 27.9 (18.1, 37.7) | <.0001 |
| **Bodily pain** | 45.3±26.0 | 64.1±27.6 | 18.8 (13.6, 24.0) | <.0001 |
| **General health** | 31.0±14.0 | 36.6±19.9 | 5.6 (1.8, 9.3) | 0.0045 |
| **Vitality** | 21.7±13.0 | 31.0±22.5 | 9.2 (4.1, 14.4) | 0.0007 |
| **Social functioning** | 67.3±27.6 | 83.7±22.0 | 16.3 (9.0, 23.7) | <.0001 |
| **Role-Emotional** | 52.6±45.4 | 64.1±42.1 | 11.5 (-1.7, 24.8) | 0.0861 |
| **Mental health** | 60.9±21.2 | 70.5±19.9 | 9.5 (6.7, 12.3) | <.0001 |
| **Physical component summary** | 29.0±7.2 | 36.0±10.6 | 7.0 (5.1, 8.8) | <.0001 |
| **Mental component summary** | 44.1±12.4 | 47.9±11.6 | 3.8 (1.4, 6.3) | 0.0023 |
| Values mean ± SD. P<0.05 based on paired t-test | | | | |

*Orthostatic Tolerance 10-minute Stand Test*

Within the ET group, the change in heart rate with standing was 4 bpm less falling from 31±14 to 27±10 bpm on the follow-up visit [-3.6 (-7.3, -0.0) p=0.0478, baseline vs. 3-months post]. Additionally, there were three individuals in the ET group who on their baseline visit were unable to stand for the entire 10 minutes, but all participants stood for 10 minutes on the 3-month follow-up visit.
